# Meso-Rex bypass shunt vs. transposition shunt for cavernous transformation of portal vein in children

**DOI:** 10.3389/fped.2022.935828

**Published:** 2022-09-07

**Authors:** Yong Lv, Lihui Pu, Jiulin Song, Jian Yang, Guoyou Zou, Jiayin Yang, Bo Xiang, Shuguang Jin

**Affiliations:** ^1^Department of Pediatric Surgery, West China Hospital, Sichuan University, Chengdu, China; ^2^Department of Critical Care, West China Hospital, Sichuan University, Chengdu, China; ^3^Liver Transplantation Center, Department of Liver Surgery, West China Hospital, Sichuan University, Chengdu, China

**Keywords:** Meso-Rex bypass, Meso-Rex transposition, cavernous transformation of portal vein, pediatric, surgery

## Abstract

**Background:**

Cavernous transformation of the portal vein (CTPV) causes portal hypertension in children. Among Meso-Rex treatments, it is unclear whether the Meso-Rex bypass shunt (MRB) or the Meso-Rex transposition shunt (MRT) offers lower postoperative morbidity. Our objective was to evaluate postoperative outcomes, comparing MRB and MRT for children with CTPV.

**Methods:**

A retrospective study was conducted on children undergoing Meso-Rex for CTPV from January 2010 to December 2020. The primary outcome was shunt complications, including shunt stenosis and thrombus. The secondary outcome was re-operation.

**Results:**

Of the 43 patients included, 21 underwent MRT and 22 underwent MRB. MRT was associated with a higher rate of shunt complications when compared to MRB (23.8 vs. 9.1%, *p* = 0.191). The patients exhibited a higher rate of re-operation under the MRT than under the MRB (19 vs. 4.5%, *p* = 0.138). The operative time in the MRT group was significantly shorter than in the MRB group. Compared to MRT, the reduction in the length and thickness of the spleen was significantly greater in the MRB group. The increases in platelets were significantly higher in the MRB group than in the MRT group. The postoperative shunt velocity of MRB was notably faster than MRT. There was no significant difference in postoperative portal pressure between the two groups (*p* > 0.05).

**Conclusion:**

Both MRB and MRT result in acceptable postoperative outcomes, but MRT is associated with higher post-shunt complications, which often increase the re-operation rate. This study suggests that MRB may offer advantages for children with CTPV.

## Introduction

Cavernous transformation of the portal vein (CTPV) is a pathological condition in which the portal vein becomes obstructed due to congenital or acquired factors (1). CTPV is prehepatic portal hypertension, which is common in children and accounts for about 40% of portal hypertension in children (2). The persistent portal hypertension results in the formation of a large number of dilated and tortuous collateral vessels around the portal vein (3). The clinical symptoms of CTPV are insidious, and the patient has no special discomfort. As the disease progresses, patients develop splenomegaly, hypersplenism, and gastrointestinal bleeding; approximately 10% of children with CTPV die from recurrent upper gastrointestinal bleeding and shock (4).

In 1992, de Ville de Goyet et al. reported the Meso-Rex procedure, in which they reconstructed a vascular conduit from the superior mesenteric vein-splenic vein junction to the intrahepatic left portal vein branch (5). The Meso-Rex procedure restores blood flow to the liver, conforms to physiological perfusion, and has become the treatment of choice for CTPV (6). In addition, the recent international symposium has shown that the Meso-Rex procedure has an important preventive effect on variceal hemorrhage in children with CTPV, making the Meso-Rex procedure an ideal treatment for CTPV (7).

The classical Meso-Rex bypass shunt (MRB) requires excision of the internal jugular vein as the graft vessel for the bypass. This procedure is more invasive, increases the number of surgery incisions, and has the potential for brain complications (8). With the promotion of this procedure and further research, a large number of studies on the modified Meso-Rex procedure have emerged, and the one that is currently used is the Meso-Rex transposition (MRT) shunt (9). Proximal splenic-left intrahepatic portal shunt (10), anastomosing inferior mesenteric vein to the left portal vein (11), the autogenous coronary and splenic vein graft (12), and the left gastric-left portal shunt (13) are among the Meso-Rex modifications. The modified Rex procedure involves a single anastomosis, streamlining the steps and reducing the difficulty and risk of the procedure. Both the classic MRB and the modified MRT procedures are increasing year by year, and both operations aim to prevent variceal bleeding and seem to offer desirable outcomes for children with CTPV. However, fewer studies comparing these two surgical approaches and previous trials have failed to reveal a clear advantage of the one technique. The surgical decision to proceed with MRT vs. MRB is a topic of much debate. Therefore, further reliable data are needed to reveal the advantages of these surgery and to provide some assistance in the design of individualized surgical protocols, which ultimately lead to the best long-term outcome for children with CTPV. The aim of the present study is to compare postoperative outcomes of a cohort of children undergoing MRB or MRT to determine if one is preferable over the other.

## Materials and methods

### Patients

The Institutional Review Board approved this retrospective analysis of consecutive patients operated on for CTPV from January 2010 to December 2020 at Sichuan University West China Hospital. Written informed consent to participate in this study was provided by the participant's legal guardian. The indication for Meso-Rex is (1) children with documented variceal hemorrhage who have progressive or continued esophageal variceal bleeding despite endoscopic intervention and who have preserved hepatic synthetic function. (2) children who fail endoscopic treatment and have intrinsic liver disease but have adequate liver synthetic function to predict that liver transplantation will not be needed for several years. (3) children with severe portal hypertension who reside a great distance from emergency medical care, endangering their survival should significant hemorrhage occur; and (4) children with extrahepatic portal hypertension and uncontrolled hypersplenism. Inclusion criteria were elective Meso-Rex surgery for CTPV (the diagnosis of CTPV was made based on imaging techniques) and age lower than 18 years. Exclusion criteria were obstruction of the left intrahepatic portal vein, the umbilical portion of the left portal vein not accessible, the occlusion of the Rex recessus, a lack of research authorization, and emergency surgery. All operations were performed by pediatric surgeons from the same team. The primary outcome evaluated was the difference in shunt complications (shunt thrombosis or stenosis) rates after Meso-Rex for children with CTPV. Secondary outcomes were demographics, perioperative laboratory examination results, gastroscopy results, ultrasonography of the liver and the spleen, re-operation rates due to shunt thrombosis or stenosis, and any additional postoperative complications according to the Clavien-Dindo classification (14). Esophagogastric varices were defined according to the classification proposed by the Japan Society for Portal Hypertension and assessed for patients undergoing Meso-Rex surgery (15). For comparison purposes, we assigned a score of 0 for 0 grade, 1 for 1 grade, and so on.

### Operative technique

**Figure 1:**
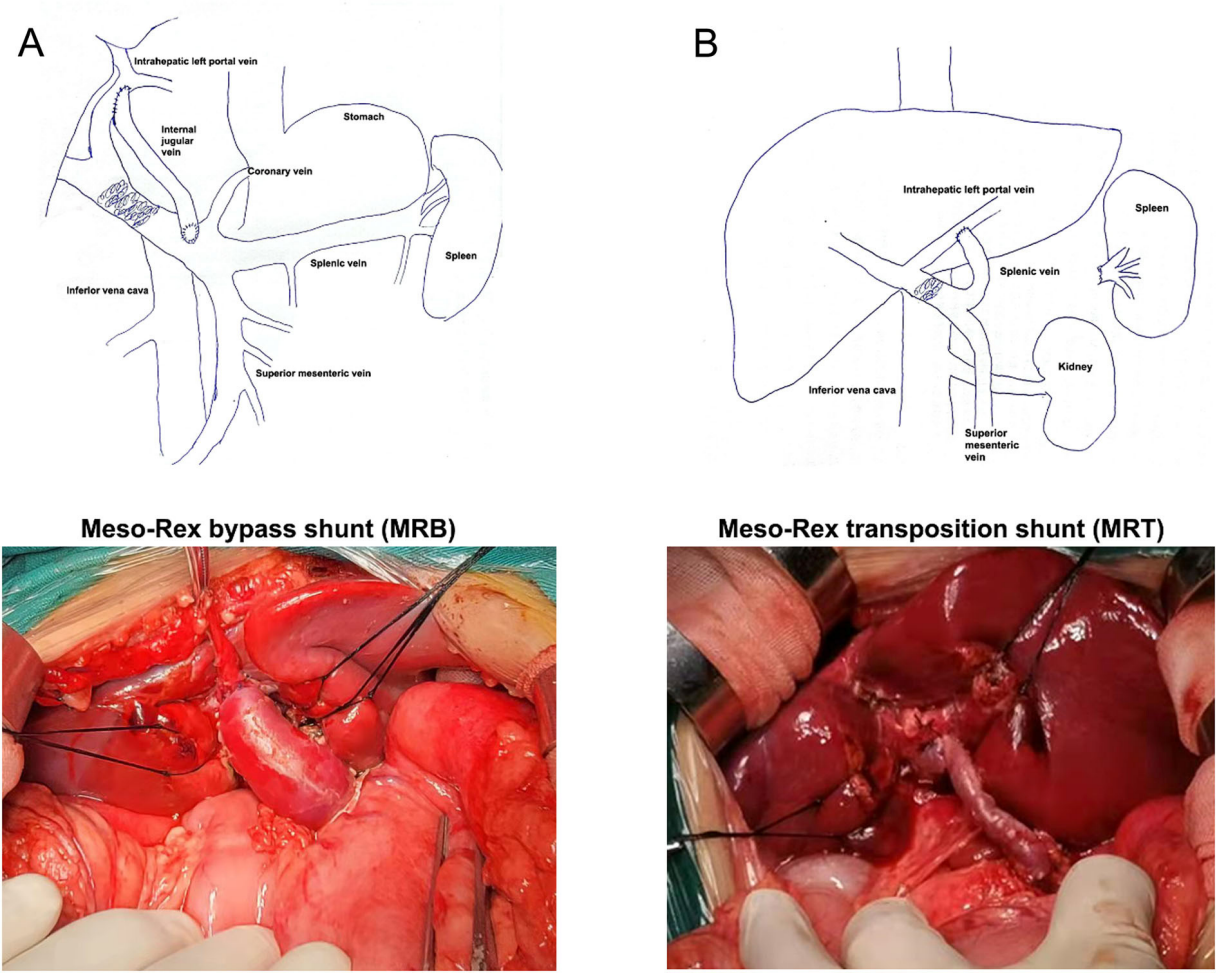
Schematic illustration of a Meso-Rex shunt. MRB, an internal jugular vein, was interposed between the superior mesenteric vein and the left portal vein within the Rex recessus of the liver.MRT, a dilated and mobilized splenic vein, was directly anastomosed to the left portal vein without the vein graft. **(A)** Meso-Rex bypass shunt. **(B)** Meso-Rex transposition shunt.

MRB was defined as the operation to connect the Superior mesenteric vein (SMV) to the intrahepatic left portal vein (ILPV) at the Rex fossa using an autogenous vein, such as an interposition jugular venous graft. MRT was defined as the operation that restores intrahepatic portal perfusion by transposition of the dilated coronary vein (Left gastric vein) or the splenic vein, and the vessel is anastomosed directly to the left branch of the intrahepatic portal vein (Figure 1). The Meso-Rex procedure begins with gastroesophageal devascularization. The terminal superior mesenteric vein is then exposed and mobilized. The portal vein pressure is measured using a manometer attached to the superior mesenteric vein cannula, and the pressure is measured in centimeters of water (cmH_2_O). The Rex recessus is exposed along the path of the hepatic round ligament. The sagittal portion of the left portal vein is exposed to identify whether it is open, occluded, or dysplastic. Then, Rex shunt construction consists of an interposition vascular conduit from the SMV-splenic vein junction to the ILPV. In the MRB group, the lateral internal jugular vein (IJV) was separated, first anastomosed end-to-end with the intrahepatic left portal vein (ILPV), and then placed in front of the duodenum to connect to the SMV in an end-lateral manner. In MRT with the splenic vein, the splenic vein is separated from the pancreas to the inferior mesenteric vein junction, transected at the hilum of the spleen, and then brought to the intrahepatic Rex recess from above the neck of the pancreas.

### Perioperative management and follow-up

The child was given broad-spectrum antibiotics within 72 h after surgery to prevent infection, and diet can be restored once bowel function is recovered. Heparin was given subcutaneously at 100 IU/(kg-d) for 7 d. Aspirin at 7 mg/(kg-d) and dipyridamole at 14 mg/(kg-d) were given orally for 6 months. Postoperative ultrasound examination was performed weekly for 1 month, every other week for 2 months, and then every 6 months to observe the patency of the anastomotic vessels to note the presence of thrombosis and the size of the spleen. After discharge from the hospital, follow-up was performed every 3 months, and blood routine, liver function, and coagulation function were regularly reviewed.

### Statistical analysis

Continuous variables were described as mean, standard deviation (SD) or median as appropriate, and categorical variables as frequencies and percentages. Significant differences between the MRB and MRT groups were tested by Fisher's test or the Chi-square test for categorical variables and the Student *t*-test or the Mann–Whitney *U* test for continuous variables. All tests were 2-sided, and a *P*-value of < 0.05 was considered statistically significant. Analysis was performed using R software (Version 4.1.0).

## Results

### Perioperative results

Of the 43 included patients, 21 (48.8%) underwent MRT, and 22 were (51.2%) treated with MRB. Table 1 reports demographics and specifics on shunt type and perioperative results. The median age at surgery was 7.6 years (range, 3.8–11.1 years). The patients treated with MRT had a shorter operative time than those treated with MRB (361 vs. 624 min; *p* = 0.001). The two groups were similar in respect to age, gender, prior abdominal surgical history, gastroesophageal varix grade, spleen size, and preoperative platelet count.

**Table 1:** Baseline characteristics for children with Cavernous transformation of the portal vein (CTPV).

	**Overall**	**MRB**	**MRT**	***P*–value**
	**(*N* = 43)**	**(*N* = 22)**	**(*N* = 21)**	
**Age(month)**				
Mean (SD)	95.7 (26.9)	96.3 (35.1)	95.0 (15.0)	0.875
**Gender**				
Female	23(53.5%)	12(54.5%)	11(52.4%)	
Male	20(46.5%)	10(45.5%)	10(47.6%)	0.887
**Gastroesophagealvarixgrade**				
Median [Min, Max]	1.00 [1.00, 3.00]	1.00 [1.00, 3.00]	1.00 [1.00, 3.00]	0.901
**Platelet count,10** ^**9**^ **/L**				
Median [Min, Max]	76.7 [51.6, 121]	74.4 [51.6, 121]	80.3 [61.7, 104]	0.225
**Spleen length, cm**				
Mean (SD)	15.4 (1.19)	15.3 (1.08)	15.6 (1.29)	0.309
**Spleen thickness, cm**				
Mean (SD)	4.41 (0.54)	4.53 (0.58)	4.28 (0.47)	0.132
**Abdominal surgical history**				
Absent	36(83.7%)	19(86.4%)	17(81%)	
Present	7(16.3%)	3(13.6%)	4(19%)	0.631
**Operative time, min**				
Mean (SD)	496(144.1)	624 (57.6)	361 (52.3)	0.001
**Shunt vascular**				
Internal jugular vein	10 (23.3%)	10 (45.5%)	0 (0%)	
Left gastric vein	12 (27.9%)	4 (18.2%)	8 (38.1%)	
Splenic vein	18 (41.9%)	8 (36.4%)	10 (47.6%)	
Inferior mesenteric vein	3 (7.0%)	0 (0%)	3 (14.3%)	0.996

### Postoperative results

The postoperative outcomes are shown in Table 2, and the median follow-up was 64 months. The shunt thrombosis rate was 4.5% in the MRB group and 14.3% in MRT. The MRT was associated with a higher shunt stenosis rate when compared to MRB (9.5 vs. 4.5%). The patients undergoing MRB had a lower overall shunt complications rate (9.1 vs. 23.8%) than the patients who received MRT, but there was no statistically significant difference noted between the two groups. There were 4/21 children with MRT (19.1%) that required re-operation vs. 1/22 children with MRB (4.5%). Of the children with MRT that required re-operation, two patients underwent a distal splenorenal shunt (Warren) for recurrent gastrointestinal bleeding. The other two patients underwent interventional radiology procedures for either shunt thrombosis or stenosis. Of the patient with MRB that required re-operation, the patient underwent a distal splenorenal shunt (Warren) for recurrent gastrointestinal bleeding. Portal pressure was reduced in both groups after the completion of the vascular reconstruction, but there was no significant difference in the level of portal pressure reduction. The symptoms of hypersplenism were relieved in both groups. Compared to MRT, the reduction in the length and thickness of the spleen was greater in the MRB group (*p* = 0.001). The platelet counts increased more in the patients with MRB treatment than in those who received MRT (199 ± 47 × 10^9^/L vs. 121 ± 57 × 10^9^/L; *p* = 0.001). The diameter of the shunt vessels of MRB was significantly larger compared to the MRT procedure (10.7 ± 0.63 mm vs. 7.9 ± 0.60 mm; *p* = 0.001). The postoperative shunt velocity of MRB was notably faster than MRT (18 ± 0.49 vs. 16.9 ± 0.46; *p* = 0.001).

**Table 2:** The postoperative outcomes for children with Meso-Rex.

	**MRB**	**MRT**	**Overall**	***P*–value**
	**(*N* = 22)**	**(*N* = 21)**	**(*N* = 43)**	
**Pre-operative-portal-pressure, cmH**_**2**_ **O**				
Mean (SD)	25.9 (1.54)	25.9 (1.47)	25.9 (1.49)	0.942
**Post-operative-portal-pressure, cmH**_**2**_ **O**				
Mean (SD)	16.6 (3.07)	17.0 (2.28)	16.8 (2.69)	0.635
**ΔPortal-pressure, cmH**_**2**_ **O**				
Mean (SD)	9.3 (3.20)	8.9 (3.11)	9.1 (3.13)	0.927
**ΔSpleen-length, cm**				
Mean (SD)	3.0 (0.53)	2.0 (0.59)	2.5 (0.75)	0.001
**ΔSpleen-thickness, cm**				
Mean (SD)	1.0 (0.13)	0.6 (0.15)	0.8 (0.26)	0.001
**ΔPlatelet-count,10** ^**9**^ **/L**				
Mean (SD)	199 (47.0)	121 (57.0)	161 (64.9)	0.001
**Shunt-vessels-diameter, mm**				
Mean (SD)	10.7 (0.63)	7.9 (0.60)	9.3 (1.50)	0.001
**Intraoperative-shunt-velocity, cm/s**				
Mean (SD)	12.3 (0.54)	12.2 (0.44)	12.3 (0.49)	0.686
**Post-operative-shunt-velocity, cm/s**				
Mean (SD)	18.0 (0.49)	16.9 (0.46)	17.4 (0.74)	0.001
**ΔShunt-velocity, cm/s**				
Mean (SD)	5.7 (0.84)	4.6 (0.66)	5.1 (0.92)	0.001
**Shunt-complication**				
Absent	20 (90.9%)	16 (76.2%)	36 (83.7%)	
Present	2 (9.1%)	5 (23.8%)	7 (16.3%)	0.191
**Shunt-thrombosis**				
Absent	21 (95.5%)	18 (85.7%)	39 (90.7%)	
Present	1 (4.5%)	3 (14.3%)	4 (9.3%)	0.271
**Shunt-stenosis**				
Absent	21 (95.5%)	19 (90.5%)	40 (93.0%)	
Present	1 (4.5%)	2 (9.5%)	3 (7.0%)	0.521
**Re-operation**				
Absent	21 (95.5%)	17 (81.0%)	38 (88.4%)	
Present	1 (4.5%)	4 (19.0%)	5 (11.6%)	0.138

ΔThe calculated difference of clinical variables between the pre-shunt and post-shunt.

## Discussion

Cavernous transformation of the portal vein is a vascular deformity and is more common in children than in adults. As a special group, the surgical procedure for children is different from that for adults. We also need to consider the growth and development of children, so it is very important to choose the appropriate surgical procedure (16). The current ideal surgical protocol should alleviate hypersplenism and avoid gastrointestinal bleeding while maintaining normal liver function (17). The Meso-Rex procedure meets the physiological characteristics of the child, reduces portal pressure, and ensures blood flow to the liver, reducing the incidence of hepatic encephalopathy, and, therefore, more and more hospitals are using this procedure for the treatment of CTPV (18).

The Meso-Rex procedure is divided into two types: Type I and Type II. Type I (MRB) uses an autologous vein to bypass the extrahepatic portal vein and the left portal vein, respectively, while Type II (MRT) directly anastomoses a dilated branch of the extrahepatic portal vein with the left portal vein (19). The important step of the Rex procedure is the selection of an appropriate shunt vessel. The classical Rex procedure requires an incision of the internal jugular vein as the graft vessel. The surgeon needs to operate on both the neck and the abdomen, which is more traumatic and requires not only the removal of the jugular vein, increasing the incision in the neck, but also the risk of pseudo-hemangiocephalus. The modified Rex procedure uses a suitable visceral vein to bypass the left branch of the portal vein, with only one intraoperative anastomosis, streamlining the procedure and reducing the difficulty and risk of the procedure. However, compared to the internal jugular vein, the visceral vein is thinner and has more branches, and the probability of postoperative thrombosis and obstruction increases accordingly. Our surgery team has completed 22 cases of MRB and 21 cases of MRT, respectively, and achieved initial satisfactory results. We conducted a retrospective study to summarize the clinical experience of the Meso-Rex procedure for the treatment of pediatric patients with CTPV and to investigate the results of MRB and MRT in order to determine the best surgical approach for our institution.

In our hospital, the average operative time of MRT was 361 min, which was less than that of MRB, probably due to the complicated anatomical process of the grafted vein and the easy bleeding of the abundant collateral vessels during MRB. On the premise of no significant difference in the surgical success rate, MRT has certain advantages over MRB, considering its simplicity of operation and small surgical trauma. Our study found a significant reduction in portal pressure after both procedures, but no significant difference between the two procedures, suggesting that both surgical techniques have similar success in relieving portal hypertension. The symptoms of hypersplenism, including the length and thickness of the spleen, were found to be relieved by ultrasonography for the two procedures. The splenic retraction and platelet elevation were more obvious in MRB than in MRT in our study. After surgery, the blockage of portal venous blood flow was lifted, the dilated splenic sinus was reduced, the fibrous tissue proliferation in the spleen was decreased, the monocyte-macrophage proliferation was reduced, and the platelet count increased. Daniel et al. found a significant increase in platelets in children after MRB (17). Our study is consistent with the findings of other authors. The increase in the platelet count was greater in children with MRB than in those with MRT (*p* = 0.001). The increase of the platelet level after operation further indicates that hypersplenism is relieved after Meso-Rex operation (9). The level of platelet increase after MRB was significantly higher than that in the MRT group, indicating that the effect of MRB in alleviating hypersplenism was better than that of the MRT group.

Blood pressure difference and velocity are very important for vascular patency. In our study, we found that, in MRT, the shunt vein has a long vascular path, a large angle, and a slow blood flow. Sometimes, the compression of the left inner lobe of the liver will also lead to the relative stenosis of the bypass vessel and slow blood velocity. However, the degree of vascular constriction after MRB is relatively small; the blood flow velocity is greater than MRT. Our ultrasonography showed elevated postoperative shunt diameters and flow velocities. The diameter of the shunt vessels was significantly larger after the MRB procedure compared to the MRT procedure. In our MRT procedure, the dilated branch of the portal vein is used as a bypass vessel, and its caliber is gradually reduced due to a decrease in portal pressure after clinical intervention. There is no compensatory thickening of the internal jugular vein, and the decrease in portal vein pressure has less effect on its diameter. MRT procedures result in the narrowing of the bypass vessels, which may lead to thrombosis or stenosis of the shunt vessels. On the other hand, all the patients in the MRT group utilized extrahepatic portal branches with a severely tortuous morphology intraoperatively, which may increase the risk of thrombosis. Therefore, when choosing portal vein system vessels as shunt vessels in MRT operation, we should try to select shunt vessels with a large diameter to reduce the degree of narrowing of shunt vessels after operation.

In this study, the incidence of shunt complications was 16.3% (7/43) in Meso-Rex, including 4 (9.3%) patients with shunt thrombosis and 3 (7%) with shunt stenosis. Previous literature reports a shunt complications rate of 4 to 28% (20). Shunt thrombosis occurred in 4.5 and 14.3% of the patients in the MRB and MRT groups, respectively. Shunt thrombosis is a serious complication after the Rex surgery, which can directly lead to surgical failure. Thrombosis of the shunt vessel after Meso-Rex surgery is also an important factor in prognosis (21). Sharif et al. reported a group of long-term follow-up cases over 5 years after the Rex operation, and the shunt thrombosis rate was 3.8% (22). A previous study has shown that the thrombosis rate after Meso-Rex was 14% and every patient with thrombosis required surgical revision (6). The outcome was consistent with our study. There are many influencing factors of postoperative thrombosis, technical factors are common causes of postoperative thrombosis, and good anastomosis technology is the basis for reducing complications. During the operation, the principle of vascular anastomosis should be strictly followed, and the appropriate length of vessels should be selected. The tension of the anastomosis should not be too high to avoid vascular distortion. The eversion of the intima of the anastomosis makes the inner wall of the vascular anastomosis smooth. In addition, thrombosis is also related to the condition of the child. Studies have found that low body weight and a high preoperative platelet count in children are related to postoperative thrombosis (20). Other studies have analyzed the effects of different anticoagulation regimens on postoperative thrombosis, but no difference has been found (6). Adequate portal vein blood flow is also an important factor to maintain vascular patency. The reduction of portal vein blood flow after Rex is a risk factor in portal vein thrombosis. Postoperative shunt stenosis is a relatively frequent and serious complication. The MRT was associated with a higher shunt stenosis rate when compared to MRB (9.5 vs. 4.5%). Postoperative vascular stenosis is not common after vascular anastomosis. Only a few studies have been reported on vascular stenosis after Rex. Lautz et al. reported that the incidence of anastomotic stenosis after Rex was 17.4%, and anastomotic stenosis occurred in weeks or years after Rex (23). Postoperative vascular stenosis mostly occurs at the anastomotic site, which is generally believed to be caused by intimal hyperplasia or scar formation at the anastomotic site (24). Due to the small diameter, deep location and angulation of the anastomotic stoma of the left branch of the intrahepatic portal vein, vascular complications after Rex are more likely to occur at the hepatic end of the bypass vessel. In addition, liver regeneration after hepatectomy may also cause the vessel to be squeezed and affect the patency of the anastomotic stoma. Stenosis may occur on either the mesenteric or hepatic side of the anastomosis, but complications occur more frequently on the hepatic side due to the angle of the shunt vessel to the intrahepatic portal vein (25). Bhat et al. reported nine cases of postoperative thrombosis in children with Rex, of which five cases occurred in the early stage, all occurring 1~9 days after surgery (average, 4 days), and 4 cases occurred in the late stage, occurring 5 months to 2 years after surgery (6). In our study, there were 4 children with postoperative thrombosis, 2 of whom had thrombosis in the early postoperative stage. Ultrasound found that the bypass vessels disappeared 3 months after the operation, and the children had no gastrointestinal bleeding, but splenomegasplenism and hypersplenism were not alleviated. The children underwent Warren surgery 2 years later, after which the symptoms of hypersplenism were relieved. The other two patients presented with hematemesis 2 years after Rex, and ultrasound examination suggested no bridging vessels. The Rex recessus was still unobstructed during surgical exploration, and the child received Rex surgery again. Therefore, for children who failed Rex surgery, there is still a chance to perform Rex surgery again. However, when the Rex recessus has been embolized or the fatigue marks are too serious to be used, Warren surgery is required. The Warren procedure is an effective treatment for recurrence after Rex operation. The MRT more often required re-operation to address postoperative thrombosis or stenosis in our study. In the MRB group, there was only one patient who eventually underwent re-operation. As opposed to MRT, the diameter of the shunt vessels is larger in the MRB group; there is some degree of luminal patency that is likely to be more amenable to medical therapy with anticoagulation. This could explain the lower likelihood of needing re-operation. All these findings confirm the beneficial effects of both procedures in restoring physiological hepatic blood flow and relieving symptoms of portal hypertension. The MRB technique has significant advantages in terms of postoperative prognosis compared to MRT, indicating that MRB is the optimal procedure in our study.

The present study also has some limitations. First, compared with the prospective study design, the retrospective study has limitations in the selection of MRB and MRT surgical methods, which is mainly based on the experience of surgeons. Second, small groups will lead to statistical bias. Because of the low incidence of CTPV in children, the inclusion of larger sample sizes, as well as multicenter studies, may be needed to draw convincing conclusions. Third, in order to reduce the difference between observers and the inaccuracy of ultrasound examination, all cases were evaluated by the same experienced ultrasound doctor. However, errors in measuring spleen size or vessel velocity and diameter cannot be completely avoided.

## Conclusion

The MRT and MRB for the treatment of CTPV in children have satisfactory outcomes and should be decided based on the condition of a child and the portal venous system vessels. MRB was associated with lower shunt complications when compared to MRT and required fewer re-operation. The splenic retraction and platelet elevation were more obvious in MRB than in MRT. Through comprehensive comparison, we recommend the MRB as the first choice for the treatment of CTPV in children. Adequate preoperative evaluation and individualized design of the surgical plan are necessary to achieve the best postoperative outcome for children with CTPV.

## Data availability statement

The original contributions presented in the study are included in the article/supplementary material, further inquiries can be directed to the corresponding author.

## Ethics statement

The studies involving human participants were reviewed and approved by West China Hospital of Sichuan University Biomedical Research Ethics Committee. Written informed consent to participate in this study was provided by the participants' legal guardian/next of kin.

## Author contributions

SJ: study concept and management and coordination responsibility for the research activity planning. YL: data analysis and drafting of the manuscript, implementation of the computer code, and support in algorithms. LP: study design and critical revision of the manuscript for an important intellectual concept. JS: data collection and the investigation process. JiayY: critical revision of the manuscript for an important intellectual concept. JianY and GZ: critical advice on study design and support for this project. BX: management and coordination responsibility for the research activity planning. All authors contributed to the article and approved the submitted version.

## Conflict of interest

The authors declare that the research was conducted in the absence of any commercial or financial relationships that could be construed as a potential conflict of interest.

## Publisher's note

All claims expressed in this article are solely those of the authors and do not necessarily represent those of their affiliated organizations, or those of the publisher, the editors and the reviewers. Any product that may be evaluated in this article, or claim that may be made by its manufacturer, is not guaranteed or endorsed by the publisher.
